# Three ways social identity shapes climate change adaptation

**DOI:** 10.1088/1748-9326/ac36f7

**Published:** 2021-11-24

**Authors:** Jon Barnett, Sonia Graham, Tara Quinn, W Neil Adger, Catherine Butler

**Affiliations:** 1 School of Geography, University of Melbourne, Melbourne, Australia; 2 School of Geography and Sustainable Communities, University of Wollongong, Wollongong, Australia; 3 Institut de Ciència i Technologia Ambientals, Universitat Autonoma de Barcelona, Bellaterra, Spain; 4 Department of Geography, University of Exeter, Exeter, United Kingdom

**Keywords:** continuity, flood, sea-level rise, self-efficacy, self-esteem

## Abstract

Adaptation to climate change is inescapably influenced by processes of social identity—how people perceive themselves, others, and their place in the world around them. Yet there is sparse evidence into the specific ways in which identity processes shape adaptation planning and responses. This paper proposes three key ways to understand the relationship between identity formation and adaptation processes: (a) how social identities change in response to perceived climate change risks and threats; (b) how identity change may be an objective of adaptation; and (c) how identity issues can constrain or enable adaptive action. It examines these three areas of focus through a synthesis of evidence on community responses to flooding and subsequent policy responses in Somerset county, UK and the Gippsland East region in Australia, based on indepth longitudinal data collected among those experiencing and enacting adaptation. The results show that adaptation policies are more likely to be effective when they give individuals confidence in the continuity of their in-groups, enhance the self-esteem of these groups, and develop their sense of self-efficacy. These processes of identity formation and evolution are therefore central to individual and collective responses to climate risks.

## 1. Introduction

Identity is the emotionally significant self-image of an individual which is derived from their membership of social groups, often referred to as in-groups (Tajfel 1974). A positive identity is the product of confidence in the continuity of in-groups, and a sense of self-efficacy (belief in capacity to exert control), distinctiveness, and self-esteem (sense of worth) (Breakwell 2015). It is a key factor in individual behaviour as people seek to act in ways that are consistent with the norms of their in-groups.

People are aware of and sensitive to threats to their identity, which can arise from changes in social or environmental circumstances, or through labelling by others. Faced with such threats, individuals can respond in several ways, including by changing their behaviour, adapting their identity, denying the existence of the threat, or accepting the existence of a threat but not otherwise changing their identity or behaviour (Breakwell 2010, Jaspal *et al* 2014).

Identity has been highlighted as an important factor in many environmental domains, including on ethical consumption, pro-environmental behaviours, climate change activism, leadership of environmental change initiatives, and sharing of information about sustainability (Whitmarsh and O’Neill 2010, Jaspal *et al* 2014, Unsworth and Fielding 2014, Sapiains *et al* 2016). Emerging research suggests that social identity, and its sub-dimensions of distinctiveness, continuity, self-esteem, and self-efficacy, can also affect climate change adaptation (Frank *et al* 2011, Fresque-Baxter and Armitage 2012, Quinn *et al* 2015, Wernersson 2018, Ruoso 2019). We therefore posit three key mechanisms through which identity formation and climate adaptation interact. Specifically, social identities can be an objective of adaptation, they can change in response to climate change information, and they can constrain or enable adaptive action. The dimensions of relationships between identity and adaptation are outlined in table 1.

**Table 1.** Critical issues on the relationships between identity and adaptation and relevant theoretical approaches.

Identity is the emotionally significant self-image of an individual that is derived from their membership of in-groups.	
Research question	Theoretical proposition
How does climate change threaten identities?	Identities may be threatened by: potential or direct experiences of climate impacts, or climate change policies, which put at risk the continuity or distinctiveness of in-groups, and senses of self-esteem and self-efficacy.
How do identities respond to climate threats?	Identities may respond by: denying the existence of climate change threats; accepting the need for behavioural and policy change; transformation through changes to in-group and/or self-images.

Maintaining and defending identity can be an objective of adaptation, for example among Indigenous groups who are struggling to hold tight to place-based and long-lived cultures that distinguish them from Western and settler cultures (Frank *et al* 2011, Rotarangi and Stephenson 2014, Eakin *et al* 2019). In cases where communities are tied to specific localities, social constructions of place, or place identity, include the identity of people that belong to them and so place identity has a strong influence on identity (Adger *et al* 2011). Nevertheless, social identities are not necessarily based on place, identities are intersectional in that they arise in response to multiple social constructions of difference (such as class, gender, nationality, and race), and this transpires at the collective (in-group) level as well as the individual level.

Maintaining identity is also critical to responses to climate impacts among groups whose occupational identity is tied to natural resources and management practices (Warner *et al* 2015, Hyland *et al* 2016), such as fishers (Coulthard 2009), farmers (Ruoso 2019), ranchers (Murphy *et al* 2017) and pastoralists (Wernersson 2018). The observation that experience of environmental change is a good predictor of adaptive behaviours may be explained by understanding those experiences as threats to identity that stimulate adaptive responses (Demski *et al* 2017).

There is some evidence that identities are responding to exogenous framings of in-groups as being more or less vulnerable to climate change (table 1). For example, people from low-lying islands framed as future climate migrants have shifted towards understandings of self that are more fixed in place or towards identities that are oriented towards transnational mobility (Farbotko *et al* 2016). The identity of places and the people tied to them may also be changing in response to climate impacts (Brown *et al* 2011). Nightingale (2017) shows how adaptation policy intersects with identity politics in Nepal, with political framings of diverse groups as being similarly vulnerable mapping onto the positions of political parties impeding recognition of the particular needs of local actors. Thus, because identity is intersectional, the response of identities to climate change is shaped by more than climate change’s materiality, but also in response to the discourses of vulnerability and adaptation that redefine the positions of subjects and shape their entitlements to resources (Eriksen *et al* 2015, Nightingale 2017).

Identities are further implicated in enabling and constraining adaptation. Adaptation policies and practices receive greater support when climate change is portrayed as a threat to issues that are important for identity and when information about climate risk and responses is exchanged between groups with similar identities (Frank *et al* 2011, Sapiains *et al* 2016, Eakin *et al* 2019). By contrast, adaptation policies and practices that threaten the continuity of Indigenous and local identities are often strongly resisted (Mortreux and Barnett 2009, Neilsen and Reenberg 2010, Eriksen *et al* 2015). Similarly, dramatic messages of climate induced catastrophe threaten people’s desire for continuity, control, and self-efficacy, and so are often denied or minimised, especially when no information about ameliorating behavioural change is available (O’Neill and Nicholson-Cole 2009, McCright and Dunlap 2011, Jaspal *et al* 2014).

There is mixed evidence about the effects of identifying with proximate and distant groups on support for adaptation policies and practices. Some studies have shown how a strong place identity can inhibit recognition of local environmental changes, and erode the legitimacy of adaptation at local scales (Bonaiuto *et al* 2002, Quinn *et al* 2019). Other studies have highlighted that sharing a sense of common cause with distant others who are risk from climate change can stimulate changes in individual behaviours and a sense of solidarity (Fresque‐Baxter and Armitage 2012, Devine-Wright *et al* 2015, Adger *et al* 2017).

Understanding how climate change affects identity, and identity in turn affects climate change responses is, we suggest, a critical knowledge frontier, and there is a strong call for more empirical research to further understanding of these connections (Fresque‐Baxter and Armitage 2012, Adger *et al* 2013, Eakin *et al* 2019). Table 1 therefore outlines the key research questions and associated theoretical propositions that help guide this contribution as well as further research.

## 2. Identity and adaptation in two vulnerable communities

We provide answers to the research questions in table 1 by synthesising findings from two detailed datasets about the responses of individuals to immediate risks from flooding and subsequent policy action in Somerset county (UK), and to long-term changes in sea-level and associated policy responses in Gippsland East (Australia). As explained below, these studies were conducted independently, but the studies have common characteristics that make synthesis across them meaningful. Both studies were conducted by social scientists who have a shared understanding of the social dimensions of climate change adaptation (e.g. Adger *et al* 2013, 2017, Quinn *et al* 2015, Graham *et al* 2018), and both used a similar methodology (see below). Further, in both places, flooding is the principal hazard, both are rural communities, and climate change is the overarching discursive and governance frame through which flooding has been understood. We do not claim that these results can be generalised across climate risks in all locations, not least because both studies were of communities in high-income countries. Nevertheless, our comparative synthesis of in-depth interview and focus group data from these two studies enables identification of the common and distinct elements of identity that are threatened across these different cases, and the responses of varied identities to different climate threats.

In Somerset, flood-affected residents were interviewed in September–October 2014 (6 to 8 months after the floods) and in April–May 2015. In the 1st round, 35 residents were interviewed (P1), and a subset of 25 residents took part in the 2nd round (P2). Interviews were conducted with residents who had been flooded in their homes directly, and those living in affected villages but who had not been inundated in their homes. Interview questions focused on residents’ relationships with the place where they live, and how social and place relationships were affected by flooding. Stakeholders with a professional interest in flood risk management were also interviewed (*n* = 52) (for more details see Butler *et al* 2018).

In Gippsland East, two sets of face-to-face semi-structured interviews were conducted. Between November 2010 and March 2011, interviews were conducted with 30 key stakeholders with an interest in adaptation to sea-level rise, including policy makers, members of key community groups, and local government representatives. The key stakeholder interviews included questions about climate change risks of concern, with a focus on understanding people and places vulnerable to sea-level rise. In April–May 2012, interviews (I) were conducted with 42 coastal residents who had been flooded in their homes directly, and those living in affected villages but who had not been inundated in their homes. Interview questions focused on residents’ relationships to the places they live and visit, the social groups they engage with, and how social and place relationships are affected by flooding. Alongside the interviews, eight focus groups (FG) were held with residents (*n* = 49) in at-risk communities in March and April 2013. Questions in the FGs explored residents’ relationships to the places they live, their experiences with flooding and how sea-level rise may affect their relationships to these places and the people who live in them (for more details about the methods see Graham *et al* 2018).

For the analysis here, interview transcripts were analysed thematically using NVivo qualitative data analysis software. Two authors coded both English and Australian datasets. The data were analysed under a pre-determined coding framework based on the orienting concepts (Layder 2013) that constitute identity, as defined above, i.e. self-efficacy, self-esteem, distinctiveness and continuity. Data were coded according to whether it reflected in-group or out-group identity (groups that the respondent does not identify with), and whether it captured how identities changed with flooding and adaptation processes. The direct quotes reported are primarily selected to be as diverse as possible to show the range of ways identities were expressed by residents and stakeholders.

### 2.1. Floods and identity in Somerset

Somerset has a long history of living with water. The name derives from Old English meaning land of summer lakes; low lying parts of Somerset have been drained and reclaimed from seasonal wetlands and lakes over centuries. Even with this drainage, the long association with flooding in the area is documented following draining from 1400 and references to flooding from the 1700s (McEwen *et al* 2014). This history and the landscape associated with wetland areas has led to residents developing a sense of place that reflects living with water (McEwen *et al* 2014). The present population in Somerset is older than the UK national average, and many respondents in the analysis here referred to retirement plans in the area, or relocation from urban areas. The perceived rural escape alongside the topography forms part of a distinct identity of place.

Living with localised flooding is part of the landscape, culture and history of lowland Somerset, with parts of agricultural land commonly being inundated in winter. However, the nature (flooding of homes), duration and spatial extent of the floods in winter 2013/14 marked these floods as a new experience for locals. Unlike previous flooding in the area, these extensive and catastrophic floods were the subject of nation-wide media reporting, which has entangled residents in local, national and global discussions of climate change risks and responses (Demski *et al* 2017).

Interviews in Somerset suggest that the floods had two principal effects on identities. First, many respondents suggested that the floods undermined their confidence in the continuity of their local communities. One respondent explained that flooding is ‘like this big black cloud that hangs over the whole village, the whole time. I just do not see a way out of that really’ (I5 P2, Somerset). Second, the response of emergency management agencies during and after the floods was a threat to the sense of self-efficacy necessary for a positive identity: ‘one of the things that engenders fear if you like, in people and stress and all the other things, is not having any information or being condescended to by the authorities’ (I12 P1, Somerset). Fears over the loss of continuity in and control over their local communities and places of dwelling resulted in receptiveness to adaptation processes that were subsequently proposed and enacted, including residents becoming actively involved in developing adaptative actions at different scales.

The evolution of identities in Somerset in response to flooding was two-fold. First, while the floods did not transform identities, they contributed to new bases for identity formation, i.e. people began identifying with others in their communities according to whether or not they had been flooded: ‘even people a couple of doors down who were not flooded, they did not have much of a clue, really…[so] it was nice to be able to go and talk flooding with people’ (I22 P1, Somerset). Second, respondents expressed new bases of identification with others outside of their communities in similar circumstances: ‘we felt like refugees, you could empathise what refugees were like and what the situation’s like for them’ (I2 P1, Somerset).

Residents accepted the need for adaptation, which was enabled in part by these processes of identification with distant others at risk of flooding, including with those that were taking adaptive action: ‘why are not we engaging more with the Dutch who historically helped drain the land here [and are now] looking at more radical solutions?’ (I4 P1, Somerset).

### 2.2. Floods and identity in Gippsland East

The research here reveals core characteristics of community identity in the study areas in Gippsland East. Notwithstanding quite distinct social positions and associated identities within each community (see Graham *et al* 2018), across the five towns involved in the research there was shared sense of people and their ancestors having made these communities during the settler period through hard work, and that that work had produced peaceful and amenable social and natural environments (Fincher *et al* 2014, 2015). Thus there was very much a sense of shared colonial history, self-efficacy, and pride in the communities involved in the study.

Part of this identity involves controlling and living with environmental perturbations, particularly flooding (but also fire). Indeed, many towns around the Gippsland Lakes are prone to coastal and riverine flooding and these can be most disruptive. The most recent disruptive flooding occurred in 2007, with some minor flooding in 2012. Other minor coastal flooding from king tides is a semi-regular occurrence. Disruptive flooding is expected to become more frequent with climate change as sea levels continue to rise (DCC 2009). Almost all local communities are socially disadvantaged, and the region is stigmatised for being both flood prone and in decline, which conflicts with local people’s sense of pride and efficacy.

Between 2010 and 2012 the town of Lakes Entrance became a test case for planning for climate change after a state planning tribunal found that the local government had failed to take account of sea-level rise in its decisions, and restrictions on development were then imposed (Hurlimann *et al* 2014). Amid local concerns about economic and population decline, these adaptation decisions can be seen as having undermined people’s self-efficacy, and their confidence in the continuity of their local in-groups. Policy makers ‘came in with a baseball bat… They just destroyed everyone’s confidence’ (I1, Lakes Entrance). The planning decisions also undermined self-esteem of the local community: ‘[they] put a really negative spin on the town’ (I1, Lakes Entrance) and ‘the doubts about rising water levels, which are affecting planning decisions, have stopped any prospect of anybody spending any money upgrading the place’ (I2, Lakes Entrance). Similar effects on in-group identities were observed in the other communities, where planning decisions were described as ‘destroying us’ (FG1, Seaspray), ‘unsettling for everyone in the community’ (I2, Port Albert), where residents feel they have been ‘blacklisted’ (I2, McLoughlins Beach) and cannot move on in their life ‘because of everything that’s been publically said’ (I1, Manns Beach).

Yet the decisions did not undermine self-efficacy. A principal response to the threat to local continuity in all five towns was the construction of narratives of resilience to flooding. For example, respondents said ‘it was happening when our grandfathers were here… it is not a new phenomenon’ (I3, Lakes Entrance) and ‘if I get flooded out, so be it… you let it flood… I am not stressed about the floods’. (I1, McLoughlins Beach). Others stressed the importance of flooding to the local environment, and normalized it, saying ‘I find floods very interesting, quietly entertaining events’ (I5, Lakes Entrance), and ‘I love the floods… it just brings all this beautiful fresh water down into the lakes’ (FG3, Lakes Entrance).

Another response of people was to identify with other groups in Victoria similarly at risk but not subject to planning restrictions. For example, respondents referred to a wealthy Melbourne suburb saying that ‘the risks that face towns like Lakes Entrance, are no different to the risks that face St Kilda esplanade’ (KS1, Lakes Entrance). Through these logics most respondents effectively denied the threat of flooding to the continuity of communities, and in so doing rejected the need for adaptation: ‘we do not need all this crap that they sit there and put on us. They do not live here. They do not see what is going on here. It does not flood. It does not do all these things. It is just a normal place’ (FG1, Port Albert).

Table 2 summarises how climate change impacts and adaptation processes can both threaten identities and cause them to evolve and adapt across these different locations. As we explain below, these findings have important implications for adapting to climate change.

**Table 2.** Summary of identity responses to climate change in Somerset and Gippsland East.

Identity is the emotionally significant self-image of an individual which is derived from their membership of in-groups.	
Research question	Evidence from cases
How does climate change threaten identities?	In Somerset the magnitude of flooding threatened the community’s confidence in its ongoing continuity, and the disaster response and recovery processes threated the community’s self-esteem and self-efficacy. The distinction between those who were flooded and those were not also created new bases of distinctiveness and solidarity with local and distant others.
	In Gippsland East the imposition of planning restrictions to manage flood risk threatened the community’s confidence in its continuity and its sense of self-esteem.
How do identities respond to climate threats?	In Somerset identities evolved to include flood risk as both part of the local identity within the community and increasingly with distant others, and to accept that adaptation is necessary. There was little evidence of significant change in identities.
	In Gippsland East local identities related to resilience were challenged and this resulted in the rejection of warnings of climate change risks, with locals asserting their capacity (self-efficacy) to adapt, and identifying with distant others who share flood risks but not planning responses to those risks. There is little evidence of identities evolving to accept the need for change.

## 3. Discussion

The research here provides evidence for three key mechanisms through which identity formation and climate adaptation interact, namely that: social identities can be an objective of adaptation, they can change in response to climate change information, and they can constrain or enable adaptive action. These mechanisms are evident in the specific cases of flood risk and externally-imposed government responses to future risks.

First, in both Somerset and Gippsland East cases identities are explicitly an object of adaptation. In both cases the continuity of in-groups, in-place, are threatened, by flooding (in Somerset), and by government responses to anticpated amplified flood risk due to climate change (in both Somerset and Gippsland East). The desire to maintain in-group continuity thus appears to be key to understanding community responses to adaptation imperatives, which were somewhat accommodating in Somerset in response to the experience of flooding, and somewhat resistant in Gippsland East in response to government directives.

Second, identities change in response to climate change information. In Somerset there is a long association with flooding but the 2013/14 experience exceeded and surpassed previous experiences and expectations. The increased discussion of climate change, and exchanges with agencies on future adaptation plans framed by climate change projections re-situated the perception of living with water in Somerset. In particular, respondents frequently mentioned other populations experiencing the impact of climate change, including displaced people elsewhere, and the work of Dutch engineers was mentioned in discussion of responses. This identification with distant others was used to frame the respondent’s own experience, and positioned the residents themselves withing a wider geopolitical landscape. In Gippsland East, government attempts to facilitate adaptation led to a reinforcement of the existing colonial identity in as much as historical associations and self-efficacy and self-esteem were strongly reasserted as reasons to deny that flooding was a problem and so, in turn, to resist the idea that adaptation interventions were necessary. This strong reassertion of communities being in control, and good places to live, was also stimulated by the stigmatisation of these communities as being in decline.

Third, the research here shows how identities can enable or constrain adaptation. The experience in Somerset suggests that elements of identity tied to belonging to and confidence in the local community can enable adaptation. When people observe climate threats to their in-group they appear more likely to accept the need for adaptation to sustain the continuity of their communities. This implies that interventions for adaptation can be enabled after extreme events, and through engagement processes that work with the self-efficacy and self-esteem of communities. They may also be enhanced by building a network that enhances solidarity among similarly adapting communities, through which knowledge and empathy can be shared.

The experience in Gippsland East suggests that identity can also present challenges for adaptation. When adaptation policies are not calibrated with local acceptance of climate change, communities may perceive such policies as threats to their continuity. Desire for continuity and damage to self-esteem leads to denial of the existence of climate risks, in part by stressing their resilience and by downplaying the distinctiveness of their circumstances. Adaptation processes must recognise local identities and experiences and tolerances of risk, and work with communities to find solutions that provide confidence in continuity and which build on senses of self-efficacy.

Our study has demonstrated the value of understanding climate change adaptation though a social identity lens. Such a lens includes analysis of shared place identies—which ours and other studies show are important for climate change adaptation (Cunsolo Willox *et al* 2012, Geoghegan and Leyson 2012)—but also draws attention to the way multiple constructions of social difference combine to create forms of identity that enable or impede adaptation practices (e.g. Osborne 2015). Knowledge of how social identities are at risk from climate change and from climate change responses is key to the broader endeavour of understanding the way climate change affects the human experience, because they are central to many psychosocial phenomena, including well being (Walker-Springett *et al* 2017), and mental health (Cunsolo and Elllis 2018). They are also a key motivator for of adaptive behaviours and support for or opposition to adaptation policies and programmes, and so a social identity lens can help advance knowledge of barriers and enablers to adaptation, particularly at the community level (Ensor and Berger 2009). Finally, mapping social identities can help explain degrees of cooperation within and between communities who may be expected to act collectively because they share common climate risks, but who struggle to do so for reasons of socially constructed differences based on religion, race, or class; and conversely who may be amenable to cooperation and collective action on climate change despite such social differences because they share a concern over climate change (Ide and Fröhlich 2015, Slevin *et al* 2022). Attention to differences in social identities between governance actors, such as those from government agencies or international NGOs, may also help explain enablers and barriers of adaptation policies and plans.

## 4. Conclusion

Identity can be harnessed to support the social acceptability of adaptation policies and plans. Conversely, efforts to initiate adaptation are likely to face challenges of legitimacy, to lack popular support, and to be resisted if they threaten social identities. Yet the same interventions could be highly effective in cases where maintaining social identity is recognised as a goal, and where policies enhance self-esteem and develop their sense of self-efficacy.

## Data Availability

All data that support the findings of this study are included within the article (and any supplementary files).
